# Comparing Different Suicide Prevention Measures at Bridges and Buildings: Lessons We Have Learned from a National Survey in Switzerland

**DOI:** 10.1371/journal.pone.0169625

**Published:** 2017-01-06

**Authors:** Alexander Hemmer, Philipp Meier, Thomas Reisch

**Affiliations:** 1 Department of Psychology, Hospital of Psychiatry Muensingen, Bern, Switzerland; 2 Department of Medicine, Hospital of Psychiatry Muensingen, Bern, Switzerland; 3 Department of Medicine, University Hospital of Psychiatry, Bern, Switzerland; Stellenbosch University, SOUTH AFRICA

## Abstract

The goal of the study was to compare the effectiveness of different suicide prevention measures implemented on bridges and other high structures in Switzerland. A national survey identified all jumping hotspots that have been secured in Switzerland; of the 15 that could be included in this study, 11 were secured by vertical barriers and 4 were secured by low-hanging horizontal safety nets. The study made an overall and individual pre-post analysis by using Mantel-Haenszel Tests, regression methods and calculating rate ratios. Barriers and safety nets were both effective, with mean suicide reduction of 68.7% (barriers) and 77.1% (safety nets), respectively. Measures that do not secure the whole hotspot and still allow jumps of 15 meters or more were less effective. Further, the analyses revealed that barriers of at least 2.3 m in height and safety-nets fixed significantly below pedestrian level deterred suicidal jumps. Secured bridgeheads and inbound angle barriers seemed to enhance the effectiveness of the measure. Findings can help to plan and improve the effectiveness of future suicide prevention measures on high structures.

## Introduction

The suicide rate in Switzerland decreased until the year 2000 and remains static in the range of 1'000 suicides per year. Suicide by jumping decreased in the years 1990 to 2013 from 173 to 123 per year. However, jumping from heights (ICD 10 X 80) continues to be the fourth most common suicide method in Switzerland [1]. It is a particularly lethal method of suicide, whereby the mortality rate varies largely depending on jump height and the type of surface below [2, 3, 4, 5, 6]. Suicide by jumping often traumatizes or even seriously hurts third parties [7, 8].

As in other countries (e.g. Taiwan)[9], the majority of suicides by jumping from heights in Switzerland are executed from buildings [10–11]. Still, about one third of all suicide jumps in Switzerland occurred at bridges [10–11]. In contrast to private buildings, public jump sites are better suited for suicide prevention, given that a great number of suicides are often limited to a few structures. At these hotspots, substantial suicide preventive effects can be achieved by a few prevention efforts.

Most interventions for suicide prevention on bridges are of a structural nature. Few, such as the Bern Muenster Terrace [8] focus on safety nets. However, the majority of the studies focus on barriers that hinder persons from climbing over. Examples include the Memorial Bridge in Augusta, Maine, U.S.A. [12]; the Bloor Street Viaduct in Toronto, Canada [13]; Clifton Suspension Bridge in Bristol, England [14]; the Jacques-Cartier Bridge in Montréal, Canada [15]; and the Grafton Bridge in Auckland, New Zealand [16, 17]. The barriers have reduced the number of suicides at these sites. However, these studies each focus on one specific jump site, which does not allow direct comparison of the different intervention measures. For example, Pelletier [12] and Sinyor and Levitt [13] showed that barriers with a height of 3.3 meters successfully hinder suicides. Yet, the height of a barrier is not the only criterion that contributes to the effectiveness of a structure. Some of the barriers examined tend to angle slightly inward toward their top ends [17, 15, 13].

Some interventions to prevent jumps from hotspots or other methods of suicide are not feasible for bridges. For example, Skegg and Herbison [18] and Isaac and Bennett [19] found that blocking access roads to hotspots deterred suicide jumps from them. This is not a viable measure for most bridges. King and Frost [20] found that the number of suicides by carbon monoxide poisoning in public parking lots has been reduced by installing aid signs. However, no studies exist that evaluate the effectiveness of aid signs as the sole intervention when used on bridges or other jumping sites, although they are widely installed. Glatt [21] and Zarkowski [22] demonstrated that, if in addition to aid signs, emergency helpline phones were directly available on bridges the phones were used on a regular basis. It must be noted that the regular use of emergency helpline phones should not be equated with the effectiveness of this prevention method [22]. Lester [23] showed that in combination with increased police presence, emergency helpline telephones led to a decrease in the number of suicides at the Sunshine Skyway Bridge in Florida, U.S.A.

Only a few studies concerning the efficacy of measures that ought to raise the probability of third-person interventions exist. For example, Bennewith, Nowers, and Gunnell [24] found that a combination of measures including barriers, closed-caption television (CCTV), and bridge employees monitoring the live CCTV video feed resulted in a reduction of suicide occurrences at the Clifton Suspension Bridge in the U.K. Although the number of events there has remained stable, bridge employees have significantly more often been involved than before the installation of CCTV [24].

Altogether, most publications on bridges and the safeguarding of buildings only examine particular structures and focus on whether a specific intervention can reduce suicides by jumping from heights. Only two studies include several buildings. Cox, Owens, Robinson, Nicholas, Lockley, and Williamson [25] conducted a meta-analysis to evaluate the efficacy of suicide prevention measures at hotspots. The authors concluded that structural interventions are an efficient way of means restriction. Pirkis, Spittal, Cox, Robinson, Cheung, and Studdert [26] demonstrated that despite a shift to other sites, at least 28% of all suicides by jumping within a city can be reduced by structural interventions.

Even if the overall effectiveness of structural interventions such as safety nets and barriers can be viewed as solid findings, no studies have directly compared the different measures in order to recommend the most effective for future safeguarding. The present study aims to determine which factors are the most effective by addressing questions: How high should a barrier be and how deep should a safety net be installed below the pedestrian level to prevent a significant number or all suicides by jumping? Is there further information that can be derived from our Swiss national survey on bridges and buildings?

## Methods

To achieve the goal of the study, we examined all available data of suicides throughout Switzerland at jumping sites that have been secured by structural interventions. Jumping hotspots can be physically secured by vertical barriers (e.g., fences, railing elevations) or by horizontal safety nets applied below the pedestrian level. Help signs of the “Dargebotene Hand” (corresponding to the Samaritans) or helpline phones are additional security measures that can be installed. To best examine to which extent these measures effectively prevented suicides, we implemented pre-post analyses by using Mantel-Haenszel Tests, regression methods and calculating rate ratios.

### Data Collection

#### Hotspots

No consensus can be found regarding how a jumping hotspot should be defined. Generally, a hotspot is defined as an accessible, usually public site that is known to be frequently used as a location to commit suicide [27].

The current study included all jump sites in Switzerland, at which occurred at least 0.5 suicides on average per year during any period of 10 years within the whole study period. In order to identify all Swiss hotspots, we first gathered data on all suicides by jumping from heights recorded by the Swiss Federal Office for statistics (BFS) for the years 1990–2010. More detailed data were provided by official bodies such as regional forensic institutes, cantonal and district doctors, as well as police authorities. We mapped these registered suicides to specific jump sites and were so able to make a preliminary identification of 31 hotspots. The BFS data had a publication delay of three years in contrast to the suicide data given by the above mentioned official bodies. The final analyses were carried out including data of the years 1990–2013.

#### Suicide-prevention measures

Information on the specific suicide-prevention measures executed at each jump site was provided by civil engineering offices and municipalities or obtained through on-site inspection. Interventions to prevent suicide were found at 23 of 31 hotspots. Due to the poor data quality (no exact installation date), seven jump sites where only signs with emergency numbers were attached were excluded from the analysis. An additional jump site was excluded because the structural intervention was conducted outside the specified data collection period. Hence, further analyses were undertaken on 15 jump locations.

All interventions that hinder or make jumping from structures impossible in the sense of means restriction as suicide prevention are considered structural measures. We made a distinction between vertical (barriers) and horizontal (safety nets) structural measures. Furthermore, we assessed for each secured hotspot whether a structural intervention secures the entire hotspot and impedes all jumps of 15 meters or more. This distinction was necessary because some structures are not secured in their entirety; e.g., for some buildings, structural measures have not been installed on their full length, or some bridges only have barriers installed on the road at their base. The cutoff point of 15 meters was chosen according to the recommendations of Moeller and Letsch [28] and Lapostolle et al. [5], who demonstrated that the lethality of a jump exceeds 50% above this height.

However, vertical interventions are not the only elements required for completely securing hotspots. Reisch et al. [11] advised that the head of the bridge also has to be secured (if climbing around is possible), that safety nets have to be installed more than three meters below pedestrian level, and that barriers have to have a minimum height of two meters. We additionally analyzed whether structures that fulfill all of these criteria show higher prevention rates than structures that do not fulfill these criteria.

In the analysis, we use the term *complete* if all of these criteria were fulfilled at a specific structure versus *incomplete* if any of the criteria was not fulfilled. These data were supplemented by data gathered on site visits. For example, elements like inbound angle installations of the barriers or specific places where barriers could be easily climbed over were investigated using a consistent protocol.

### Analysis

We used a pre-post analysis comparing data before and after the installation of the measure for all structures and each individual structure.

First, the suicides that occurred the years 1990–2013 were assigned to the pre- and post-intervention phases, according to when they occurred. The mean observation time recorded was 252.00 months (*SD =* 47.14 months; *Min*. *=* 156.00 months; *Max =* 288.00 months).The mean of the pre-intervention phase was 178.60 months (*SD =* 54.88 months; *Min*. *=* 48.00 months; *Max =* 264.00 months) and the mean of the post-intervention phase was 73.40 months (*SD =* 49.18 months; *Min*. *=* 24 months; *Max =* 180 months). Despite its partial safeguarding, the total construction phase of a suicide prevention measure (*M =* 7.30 months; *SD =* 7.19 months; *Min*. *=* 1 month; *Max =* 27 months) was assigned to the pre-intervention phase.

To test the overall effect of the prevention measures across jump sites, both the Mantel-Haenszel Test and maximum-likelihood methods (negative binominal regression) were calculated. Furthermore, the above-mentioned test procedures were used to include the specific type of intervention measure as a covariate in the analyses and to calculate the overall effects of the measure group *barrier* and *safety net* (negative binominal regression). Note that including the variable *extent* to the model leads, to the combination “complete and nets” with only two observations. For the variable *extent*, only confidence intervals based upon the ML-estimator and the standard error of the rate ratio were calculated. To review the effects of suicide prevention measures at individual bridges, we calculated rate ratios and built confidence intervals based on the standard error (s. e.) of the log rate ratios (log*RR*) and *p-*values based on the test statistic log *RR*/*s.e.*(log *RR*) ∼ *N* (0.1) specified. Additionally, we compared suicide reduction rates of safety nets and barriers as well as complete and incomplete interventions by using Mann Whitney-U tests.

## Results and Discussion

### Description of Analyzed Jump Sites

Hotspots are anonymized in order to minimize a possible Werther Effect analogous to Beautrais [16]. A total of 15 jump sites could be included in the present study; 13 bridges, 1 terrace, and 1 multi-story car park.

The jump sites were on average 62.94 m high (range 33.80 m to 150.00 m; *SD =* 23.00 m). The average barrier height before the suicide prevention intervention measures were installed was 1.13 m (*SD* = 0.14 m); the highest barrier was 1.30 m high, and the lowest was 0.80 m. On three bridges, the original barrier height could not be determined. On average, the jump sites were 2.75 km (*SD* = 3.71 km) away from a town center. The detailed figures for all analyzed bridges are included in Table 1.

**Table 1:** Technical Data of the Included 15 Jump Sites.

Jump site	Type of building	Prevention type	Measure complete	Height (m)	Barriers: Height of railing (m)	Net installed below pedestrian level (m)	Help sign	Additional information from site visits
A	Bridge	Barrier	YES	58	1.9	n.a.	YES	Bridgeheads secured, emergency phones, distance to city center 2.9 km, distance to psychiatric hospital 4 km
D	Bridge	Barrier	NO	23	1.51	n.a.	NO	Inward angle of the barrier, distance to city center 0.7 km, distance to psychiatric hospital 2.8 km
E	Bridge	Barrier	YES	85	1.8	n.a.	NO	Distance to city center 2.6 km, distance to psychiatric hospital 4.8 km
F	Bridge	Barrier	YES	47	3.25	n.a.	NO	Distance to city center 1.3 km, distance to psychiatric hospital 0.7 km
K	Bridge	Barrier	YES	68	2.3	n.a.	YES	Distance to city center 3.1 km, distance to psychiatric hospital 18 km
M	Bridge	Barrier	NO	75	2.65	n.a.	YES	Inward angle of the barrier, climbing around bridgeheads possible, distance to city center 1.5 km, distance to psychiatric hospital 18.2 km
H	Bridge	Barrier	YES	150	2.58	n.a.	YES	Emergency phones, distance to city center 5.5 km, distance to psychiatric hospital 5.8 km
B	Bridge	Barrier	NO	33	2.9	n.a.	YES	Distance to city center 0.8 km, distance to psychiatric hospital 3.1 km
C	Bridge	Barrier	NO	47	2.9	n.a.	YES	Distance to city center 0.7 km, distance to psychiatric hospital 3.5 km
O	Bridge	Barrier	NO	55	1.7	n.a.	NO	Distance to city center 2.1 km, distance to psychiatric hospital 2.1 km
L	Multi-story-parking	Barrier	NO	30	2.4	n.a.	NO	Only the top levels were secured, ramp not secured, distance to city center 0.6 km, distance to psychiatric hospital 1.4 km
N	Bridge	Safety net	NO	103	n.a.	0.5	YES	Width of net 4.0, distance to city center 15.1 km, distance to psychiatric hospital 2.2 km
I	Bridge	Safety net	NO	99	n.a.	4	YES	Width of net 5.2 m, distance to city center 3.4 km, distance to psychiatric hospital 4.4 km
J	Terrace	Safety net	YES	35	n.a.	7	YES	Width of net 6.0 m, distance to city center 0.8 km, distance to psychiatric hospital 3 km
G	Bridge	Safety net	YES	31	n.a.	4	NO	Width of net 5.0 m, distance to city center 0.1 km, distance to psychiatric hospital 4.2 km

Note. Bridges were anonymized in order to minimize Werther Effects.

### Description of Suicide-Prevention Measures

Of the 15 jump sites, 11 (73.3%) were secured by barriers (fences). Five (45.5%) of these jump sites have complete fences, and 6 (54.5%) have incomplete fences. On average, the security barriers have a height of 2.30 m (*SD* = 0.61 m). After the construction of the security barrier, the minimum railing height is 1.50 m, and the maximum height is 3.30 m. With one exception, all vertical barriers were raised to at least 1.70 m. Two of the fences have additional inward angles (bridges D, M). One bridge was additionally secured with side barriers on the bridgeheads in order to prevent climbing around the fences (bridge A). Six of the areas secured by fences have been additionally equipped with aid signs displaying emergency helpline numbers of the “Dargebotene Hand.” Four (26.7%) jump sites were secured by safety nets. At 2 (50.0%) sites, the nets secure the complete jump area. Two nets (50.0%) are incomplete. On average, the safety nets have a depth of 3.88 m (*SD* = 2.66 m) below street level. The minimum depth is the net on bridge N with 0.50 m, and the maximum depth is 7.00 m on terrace J. Three of the areas have been additionally equipped with aid signs displaying emergency helpline numbers (see Table 1).

### Overall Effectiveness of Jump Site Safeguarding

The Mantel-Haenszel Test, respectively the negative binominal regression of the aggregated data, of the 15 jump sites shows that the rate ratio from pre- to post-installation of structural measures (barriers and safety nets) is *RR*_MH_ = 0.32, CI_95%_ = 0.23, 0.44 resp. *RR*_GLM.NB_. = 0.3, CI_95%_ = 0.17, 0.44. This corresponds to a reduction of the occurrence of suicides by 71.7%. In the pre-intervention phase, 327 suicides were carried out during 2679 months. This corresponds to a rate of 0.12 suicides per month or 1.47 per year. In the post- intervention phase, 38 suicides occurred during 1101 months, corresponding to a rate of 0.035 suicides per month or 0.41 per year.

#### Safety nets

Safety nets led to a 77.1% reduction of suicides. The rate ratio from before to after the installation of safety nets is 0.21, CI_95%_ = 0.07, 0.62. During 656 months, 55 suicides occurred in the pre-intervention phase. This corresponds to a rate of 0.084 suicides per month or 1.00 per year. In the post-intervention phase, during 364 months, 7 suicides occurred, corresponding to a rate of 0.019 suicides per month or 0.23 per year.

#### Barriers (fences)

Aggregated data of all sites secured by fences show that this intervention led to reduction of suicides by 68.7%. The rate ratio from before to after installing the barriers is 0.34, CI_95%_ = 0.18, 0.64. In the pre-intervention phase, 272 suicides occurred during 2023 months. This corresponds to a rate of 0.13 suicides per month or 1.61 per year. In the post-intervention phase, 31 suicides occurred during 737 months (0.042 suicides per month or 0.51 per year).

#### Extent

Complete safety measures led to reduction of suicide by 82.0%. The rate ratio from before and after installing is 0.18, CI_95%_ = 0.10, 0.44. In the pre-intervention phase, 184 suicides occurred during 1360 months. This corresponds to a rate of 0.14 suicides per month or 1.62 per year. In the post-intervention phase, 23 suicides occurred during 488 months (0.047 suicides per month or 0.57 per year). Incomplete safety measures led to a reduction of suicide by 44.8%. The rate ratio from before and after installing is 0.55, CI_95%_ = 0.45, 0.86st. In the pre-intervention phase, 143 suicides occurred during 1319 months. This corresponds to a rate of 0.11 suicides per month or 1.30 suicides per year. In the post-interventions phase, 15 suicides occurred during 613 months (0.02 suicides per month or 0.29 per year).

Complete interventions were significantly more effective than incomplete safety measures (Mann-Whittney U test; p = .029). No significant difference was found between safety nets and barriers.

### Analyses of Individual Structures

The rate ratios of the individual structures show that the efficacy of the safety measures ranges from 2.1% (structure L) to 100% (structures F, H, J, & K). Bridges A, B, and D exhibit a statistically significant effect (p <. 05). Due to the absence of suicides in the post-phase, the standard errors at structures F, H, J, and K could not be calculated for statistical-methodological reasons. However, all of the latter analyses would have been statistically significant if one (instead of zero) suicides would have been observed. An overview of the effects of the prevention measures are shown overall and for individual structures in Tables 2 and 3, respectively.

**Table 2:** Reduction of Suicide Rates After Securing Jump Sites by Structural Means: Group Analysis.

Jumpsites	Measure			Suicide rate before installation of the safety measure							
	Type of structural intervention		Were all parts secured that allow lethal jumps?				Suicide rate after installation of the safety measure			Reduction of suicide rate	
	Barriers (Vertical)	Savety nets (Horizontal)		Suicides per year	Suicides observed	Months of observation	Suicides per year	Suicides observed	Months of observation	Prevention rate (%)	(RR*; CI95%)
All	YES	YES	n.a.	1.465	327	2679	0.414	38	1101	71.7	0.30; 0.17, 0.44**
All barriers (overall)	YES	NO	n.a.	1.613	272	2023	0.505	31	737	68.7	0.34; 0.18,0.64**
All safety nets	NO	YES	n.a.	1.006	55	656	0.231	7	364	77.1	0.21; 0.07, 0.62**
All structures that hinder lethal jumps	n.a.	n.a.	YES	1.624	184	1360	0.566	23	488	82.0	0.18; 0.10; 0.44***
All structures that still allow lethal jumps	n.a.	n.a.	NO	1.301	143	1319	0.294	15	613	44.8	0.55; 0.45; 0.86***

*Note*.

*RR = rate ratio.

**Test procedure: GLM, Negative binominal distribution.

*** Confidence intervals based on the ML-estimator and the standard error of the rate ratio.

**Table 3:** Reduction of Suicide Rates after Securing Jump Sites by Structural Means at Each Jump Site.

Jumpsites	Measure			Suicide rate before installation of the safety measure			Suicide rate after installation of the safety measure			Reduction of suicide rate	
	Type of structural intervention										
	Barriers (Vertical)	Safety nets (Horizontal)	Were all parts secured that allow lethal jumps?	Suicides per year	Suicides observed	Months of observation	Suicides per year	Suicides observed	Months of observation	Prevention rate (%)	(RR^1^; CI95%; p-value)
A	YES	NO	YES	3.014	54	215	0.986	6	73	67.3	0.33 ; 0.14, 0.76, p = 0.01^2^
D	YES	NO	NO	3.234	45	167	0.992	10	121	69.3	0.31; 0.15, 0.61, p = <0.01
F	YES	NO	YES	0.727	16	264	0.000	0	24	100.0	rtz^3^
H	YES	NO	YES	0.867	13	180	0.000	0	60	100.0	rtz
K	YES	NO	YES	0.733	8	131	0.000	0	25	100.0	rtz
M	YES	NO	NO	0.385	5	156	0.273	3	132	29.1	0.71; 0.17, 2.97, p = 0.64
B	YES	NO	NO	3.313	53	192	0.250	1	48	92.5	0.08 ; 0.01, 0.55, p = 0.01
C	YES	NO	NO	2.313	37	192	0.750	3	48	67.6	0.32 ; 0.10, 1.05, p = 0.06
E	YES	NO	YES	1.171	24	246	0.571	2	42	51.2	0.49; 0.12, 2.07, p = 0.33
L	YES	NO	NO	1.082	11	122	1.059	3	34	2.1	0.98; 0.27, 3.51, p = 0.97
O	YES	NO	NO	0.456	6	158	0.277	3	130	39.2	0.61; 1.15, 2.43, p = 0.48
J	NO	YES	YES	2.250	9	48	0.000	0	180	100.0	rtz
G	NO	YES	YES	0.903	14	186	0.400	1	30	55.7	0.44 ; 0.06, 3.37, p = 0.43
I	NO	YES	NO	1.205	25	249	0.923	3	39	23.4	0.77; 0.23, 2.54, p = 0.66
N	NO	YES	NO	0.486	7	173	0.313	3	115	35.5	0.64; 0.17, 2.49, p = 0.52

*Note*.

1. RR = rate ratio.

2. Confidence intervals based on the standard error of the log rate ratios.

3. rtz = Reduction to zero.

No statistical analyses can be carried out if no suicide has occurred in the post-intervention period. Therefore, no standard errors are defined, and no confidence intervals are presented.

## Discussion

The results of the current study provide empirical evidence that structural interventions such as barriers or safety nets show a preventive effect. They are consistent with previously published studies [16, 17, 14, 25, 12, 15, 26, 8, 13]. It has been unclear though if earlier meta-analyses and individual case studies exhibit a publication bias. According to Pirkis et al. [26], it cannot completely be ruled out that only results that show significant effects are published and other studies that show non-significant or counterproductive data are not released. In contrast to the mentioned studies, the current study has a pre-post design and has systematically examined all bridges with a high occurrence in suicide jumping of one country (Switzerland) and a publication bias can be ruled out. Altogether, the reduction in suicides across all jump sites represents 71.7%. The suicide rate could be reduced from 1.47 suicides per year to 0.41 suicides per year. This figure lies slightly lower than that of Pirkis et al. [26], who found a reduction of 86%. It is to be assumed that this difference can be explained by the fact that ineffective as well as only marginally effective prevention measures were also included in the present study (refer to Table 2 jump sites L, M, N, or O). For the years 1990 to 2013, suicide by jumping in Switzerland in general decreased. However, it is not possible to state definitively whether this decrease can be attributed entirely or at least partly to the interventions mentioned in the current study.

Safety nets were not statistically significant more preventive than safety barriers. Incomplete measures led to an insufficient prevention of suicides. It seems to be more important that a structural measure secures all parts of a bridge that allow lethal jumps, and it seems less important which kind of structural measure (safety net versus barrier) is chosen. More data is needed to determine whether there is in fact a difference between safety nets and barriers.

It is noteworthy that the structural intervention measures at 4 of the 15 examined jump sites led to a complete stop in suicides. These measures were safety nets at jump site J, which are fixed far below street level (7 m), have a wide overhang (6 m) and secure all areas that allow lethal jumps. At terrace J, the full reduction in suicides has been continuing for 15 years. Second, barriers that are very high (at least 2.3 m), secure the jump site across the entire length, and prevent climbing around the bridgeheads also led to a complete elimination of suicides. In the literature, only Pelletier [12] could show similar sustainable results as terrace J. Terrace J can thus be seen as the gold standard in terms of using safety nets to secure a hotspot against this type of suicide. It also seems worthy to note that the specific barrier height that led to the elimination of suicides from this hotspot is slightly lower in the present study than in the given literature [12, 13]. In regard to the low barrier height of 2.3 m at bridge K, further research will show if the termination of occurred suicides can be sustained in the future.

Which exact prevention measure was chosen for a specific structure depended on various factors. If particularly aesthetic factors [16, 17] are weighted, safety nets should be considered as the intervention measure because when seen from a distance, they clearly impair the aesthetic of buildings less than barriers. The depth of the installation of safety nets was mostly limited due to architectural reasons. Some bridge structures do not allow the attachment of safety nets below the depth of 3 m. Here, due to structural reasons, only barriers as safety measures should be chosen; otherwise, only small and inadequate prevention effects can be expected (jump site N, I or G; see Table 2). Safeguarding with safety nets is considerably more expensive compared to barriers. If primarily financial factors are considered, barriers have to be mounted.

It seems astounding that an increase in barrier height to 1.51 m of bridge D already led to a reduction in suicides of 69.3%. It can be assumed that this unexpected strong effect is connected to the inward inclination of the barrier. Thus, the inward tilted barriers at Grafton Bridge [16, 17] and at Bloor Street Viaduct [13] also led to a complete stop in suicides.

Barrier height as well as depth and width of safety nets are central, but non-exhaustive criteria in the safeguarding of constructions. Ultimately, the weakest link in the security chain seems to be crucial with regard to how effective suicide prevention interventions are. This is particularly evident in cases where bridgeheads are climbed around (e.g., bridge M). To achieve the highest possible suicide-preventive effects, bridgeheads should be secured in any case. This result may also explain why aid signs without structural changes are insufficient. They leave several weak links in the security chain that may be closed by police patrols or other measures [23].

### Limitations

Along with physical availability, psychological availability by media reports [29, 30, 31] is a decisive factor in the development and maintenance of a hotspot. Effects by media were not included in the present study. Furthermore, the study has not reviewed whether there has been a shift to nearby jump sites as a result of safeguarding a specific jump site. Previous work [14, 32, 12, 26, 8, 13, 18] has shown that the shifting effect caused by safeguarding a specific jump site is minimal or rather has even resulted in a reduction in suicides at nearby jump sites. A further limitation of this study is that in part, calculations had to be carried out with a very small number of cases. Due to the small power of the analyses, the likelihood of finding significant effects is rather small, especially in regard to analyses of individual structures.

Although we included data from several official bodies, it is possible that we missed some rare cases of suicide by jumping (e.g., the body of a person floated away in the river below the bridge). The data spanning from 1990 to 2013 do not allow statements about the time before 1990. It is possible that some early hotspots have been unrewarded. Additionally, we don`t know how the included hotspots developed before 1990. Furthermore, the date of intervention was not controllable. We had to compare different pre-post periods. Bias cannot be excluded completely. Moreover, the current study does not mention attempted suicides. It is important that additional studies confirm our findings and provide a more complete picture by including suicide attempts.

Data regarding the date of installation dates of helpline phones and most aid signs could not be determined and could therefore not be included in the statistical pre-post analyses. However, at least eight hotspots of the original sample stayed hotspots after the installation of help signs. Help signs on their own were often not sufficient to significantly reduce the number of suicides on hotspots.

### Policy and Practice Recommendations

On the basis of the these results, we recommend safeguarding jump sites with a high occurrence of suicide (at least 0.5 suicides per year) by means of barriers or safety nets. Barrier height should be at least 2.3 m, and bridgeheads should specifically be secured in addition to prevent climbing around them. Safety nets should lie significantly below pedestrian level and have a net width adapted to the depth. Based on our data, a depth of 4 m below pedestrian level may be sufficient. Safeguarding should be complete or at least not allow jumps of 15 meters or more. In part, these recommendations were incorporated into the Regulation of the Swiss Federal Road Office regarding the suicide-preventive safeguarding of bridges [11]. These recommendations should be substantiated by further empirical research and, if necessary, adjusted accordingly.

## Supporting Information

S1 Table Incidents Locations 1990–2013. (XLS)
